# Supplementary material for: A Computational Framework for Understanding the Impact of Prior Experiences on Pain Perception and Neuropathic Pain
Source: PLoS Comput Biol. 2024 Oct 31;20(10):e1012097. doi: 10.1371/journal.pcbi.1012097 (PMC11556707; doi:10.1371/journal.pcbi.1012097)
Supplement: S1 Supplementary Results — (DOCX) [file pcbi.1012097.s001.docx]

# S1 Supplementary Results

#### Fig A – Study 2 from (Jepma et al., 2018) [20] with single-layer Kalman filter

These simulations are run as described for Fig 1 in the main manuscript, with three key changes:

1. Here, we use the experimental data from Study 2 from (Jepma et al., 2018) [20], instead of Study 1 as in the main manuscript.
2. We have included a third, neutral cue (as in the experimental data of Study 2) by letting $\boldsymbol{u}^{(k)}$ be a three-dimensional vector such that $\boldsymbol{u}_{placebo}^{\left( k \right)}=\left[ u 0 0 \right]^{T}$, $\boldsymbol{u}_{nocebo}^{\left( k \right)}=\left[ 0 u 0 \right]^{T}$ and $\boldsymbol{u}_{neutral}^{\left( k \right)}=\left[ 0 0 u \right]^{T}$ and $\hat{B}=[\hat{b}_{placebo}, \hat{b}_{nocebo},\hat{b}_{neutral} ]$, where $\hat{b}_{neutral}\sim N\left( 0.9, {0.8}^{2} \right)$ (same as the initial value of the elements of $\hat{B}$ for the conditioning simulations for Fig 4).
3. We have altered the level of tissue damage elicited by the thermal stimulation to be $x_{low}=2.5$ for low heat trials (47°C, $x_{low}=3.1$ for Fig 1) and $x_{high}=3.5$ for high heat trials (48°C, $x_{high}=4.3$ for Fig 1), to reflect differing sensitivity to heat at different locations on the body. In Study 1, the thermal stimuli were applied to the inner forearm, and in Study 2 the site of stimulation was the lower leg (which is less sensitive, hence the lower values of $x$).


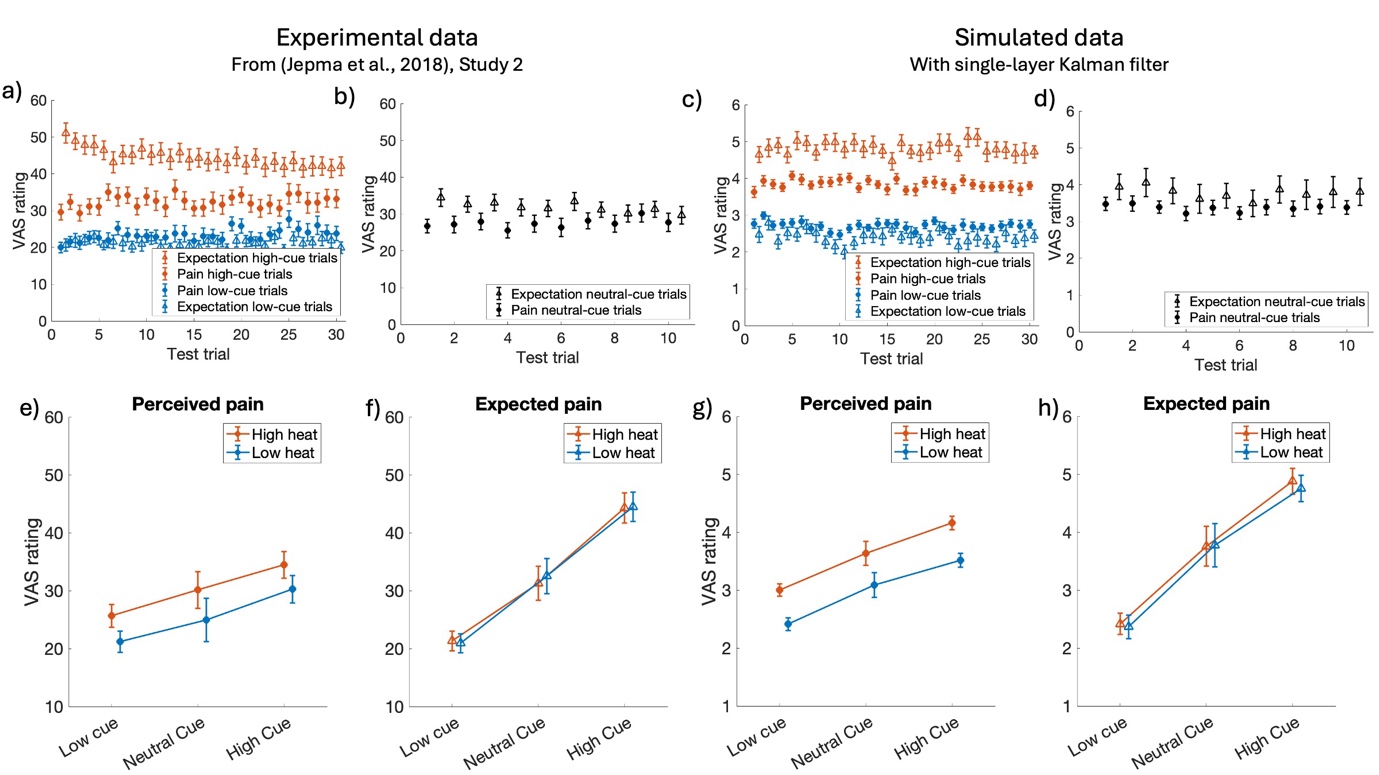


Fig A. Experimental data from Jepma et al., [20] Study 2 (left) and our Kalman filter simulations results (right). **a**), **b**), **c**) and **d**) average expected (open triangles) and perceived (filled circles) pain as a function of cue type and trial for experimental and simulated data, respectively. **e**) and **g**) perceived (filled circles), **f**) and **h**) expected (open triangles) pain as a function of stimulus temperature and cue type for experimental and simulated data, respectively. Error bars indicate inter-individual standard errors. Note that the experimental data is measured on a 100-unit scale, whereas the simulated data is on an 11-unit scale.

#### Fig B. Study 2 from (Jepma et al., 2018) [20] with hierarchical Kalman filter

These simulations are run as described for Fig 4 in the main manuscript, but with the same modifications as for Fig A.


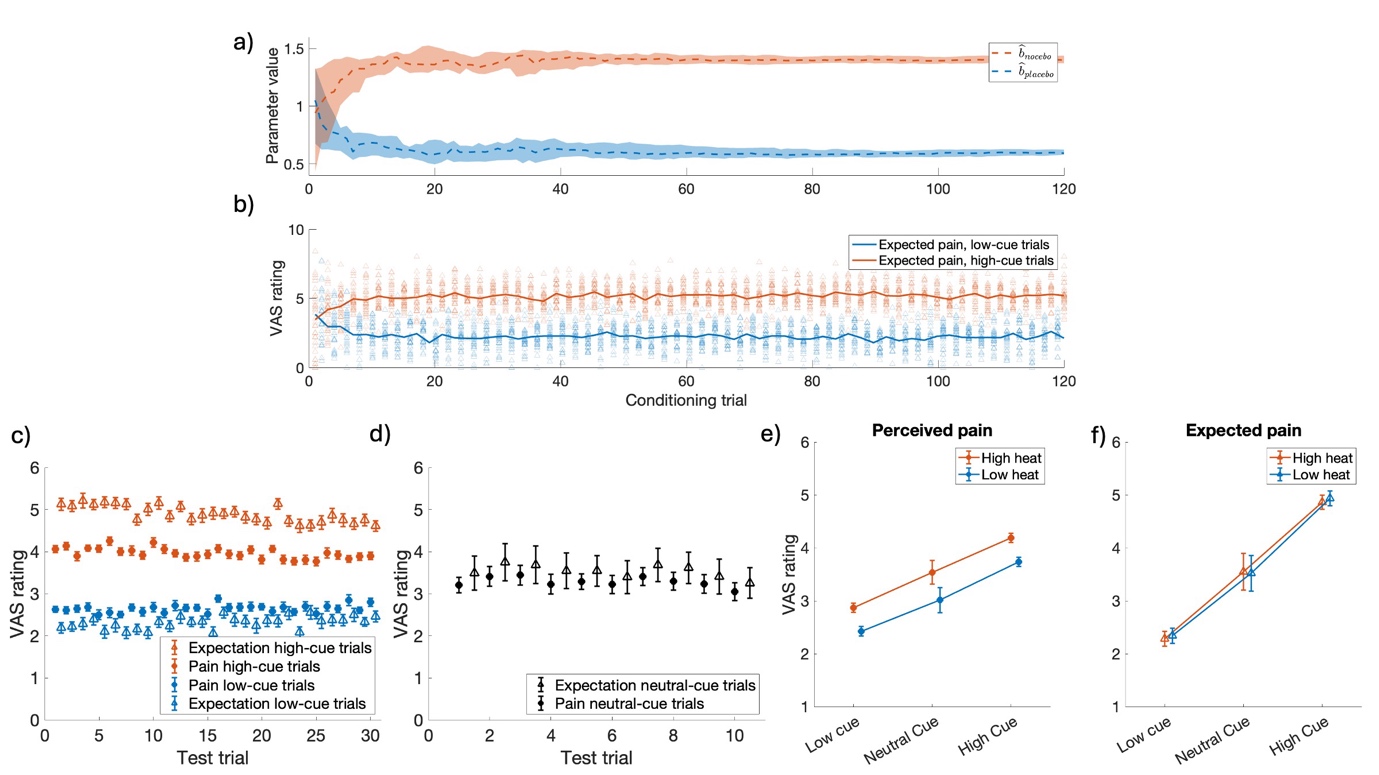


Fig B Results of the hierarchical Kalman filter simulations of classical conditioning, when using the data from (Jepma et al., 2018) [20] Study 2. In the testing phase of Study 2 an additional, neutral cue is introduced, which was not included during the learning phase of the experiment. **a**) median value of $\hat{b}_{nocebo}$ (dashed red line) and $\hat{b}_{placebo}$ (dashed blue line) across conditioning trials. Shaded areas indicate the interquartile range. **b**) average expected pain for high-cue trials (red) and low-cue trials (blue) during conditioning. Open triangles indicate the expected pain for each participant on each conditioning trial. **c**) and **d**) the average expected ($\hat{\bar{x}}$, open triangles) and perceived ($\hat{x}$, filled circles) pain as a function of cue type on each test trial. **e**) perceived (filled circles,) and **f**) expected (open triangles) pain as a function of stimulus temperature and cue type. Error bars indicate inter-individual standard errors.

#### Fig C. Chronic pain without control input

Here, we run simulations just as for Fig 2 in the main manuscript, but with $u^{\left( k \right)}=0 \forall k$. As described in the Methods section, this scenario reflects a situation where there are no predictive cues of an upcoming noxious stimuli $\tilde{u}^{\left( k \right)}$.


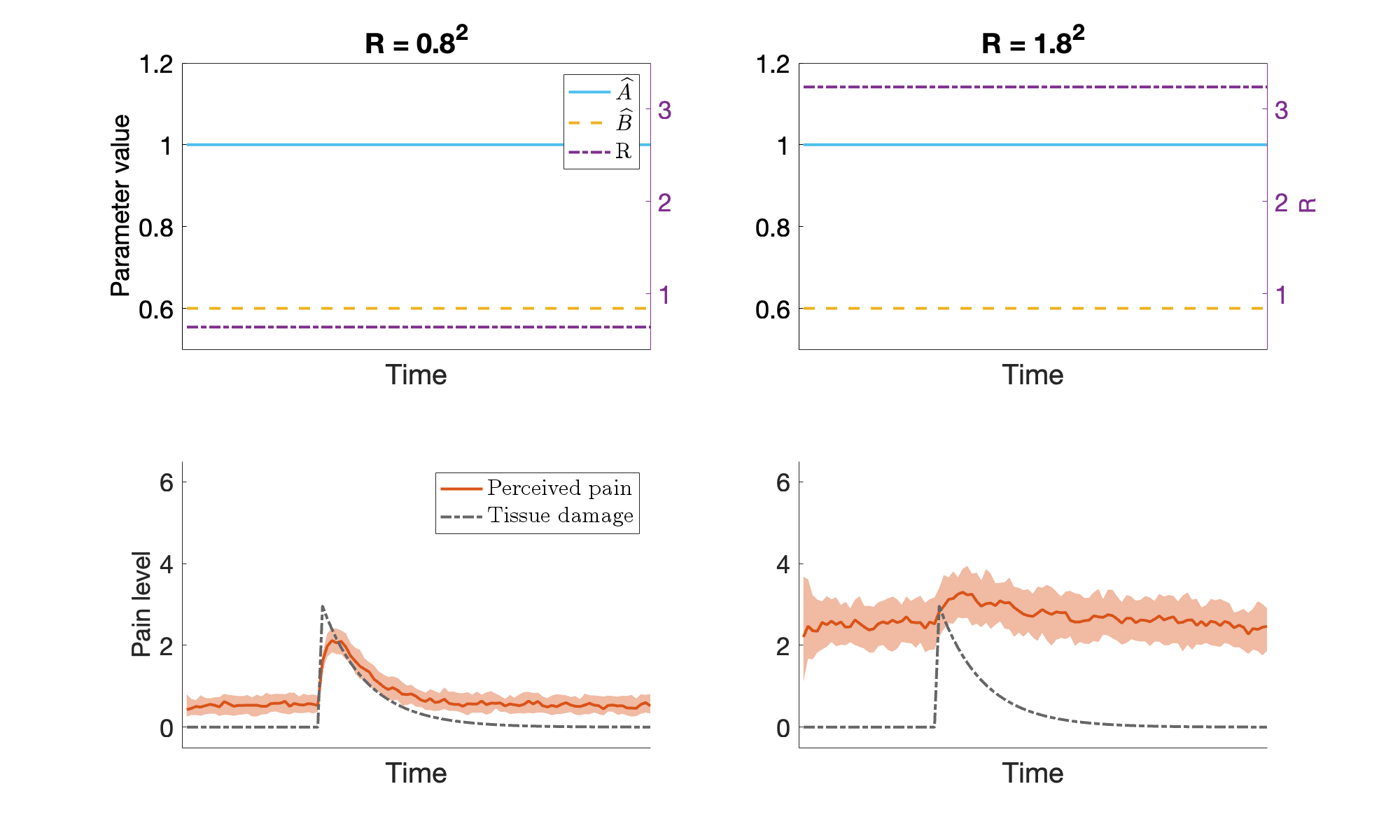


Fig C Results of the Kalman filter simulations of chronic pain with control input $u^{\left( k \right)}=0 \forall k$. The results are similar to those presented in Fig 2 in the main manuscript. Despite having no predictive cues $u^{\left( k \right)}\neq0$ of the upcoming noxious stimuli, the increase in tissue damage still gives rise to a sensory response that is sufficient to increase the level of pain. Additionally, when there is elevated uncertainty in the sensory input and the internal model parameter is $\hat{A}\approx1$, the pain may persist even after the tissue damage has recovered.
