# Supplementary material for: A Computational Framework for Understanding the Impact of Prior Experiences on Pain Perception and Neuropathic Pain
Source: PLoS Comput Biol. 2024 Oct 31;20(10):e1012097. doi: 10.1371/journal.pcbi.1012097 (PMC11556707; doi:10.1371/journal.pcbi.1012097)
Supplement: Attachment — Submitted filename: review_june_24.docx [file pcbi.1012097.s002.docx]

Dear Editors, Authors and Readers,

Authors highlight an open question in the field of pain neuroscience as to what mechanisms/processes gives rise to prior beliefs, which in turn affect the experienced level of pain, with relevance to chronic pain states but also effects such as placebo and nocebo. This is of course a very important issue as we know surprisingly little about pain inference, especially in the times where chronic pain is such a prevalent issue (20-30% of population). Authors briefly outline the relevant research to position their question within, particularly mentioning Bayesian inference as a promising way to model such phenomena. Authors themselves propose the use of a (modified) Kalman Filter (KF) model (an algorithm used to estimate latent states in a specific setup of probabilistic / Bayesian inference) to describe phenomena such as placebo hypoalgesia, nocebo hyperalgesia and neuropathic pain.

The authors indeed succeed in reproducing the stated phenomena (placebo/nocebo, neuropathic pain) through simulation. They also demonstrate their model’s predictions in the context of classical conditioning paradigm to induce nocebo/placebo effects. The results are interesting and it’s promising that the HKF model is able to capture these effects. However, in order to appreciate this, the reader is required to jump between figures captions, main texts and methods to put a story together. As such, the article will benefit from more clarity regarding modelling descriptions and results reporting (with a clearer mapping between the two). Additionally, a comparison of model predictions to a pre-existing dataset (see comment below) could provide a more convincing story. The proposed application of Hierarchical Kalman Filter in the context of pain is novel and holds promise for modelling pain inference. Additionally, it allows for some of the system parameters to be estimated online “by the participant’s sensory/cognitive system” rather than inferred post-hoc from the data, and as such is a valuable approach. However, it’s important to note that other parameters still need to be hand-tuned or estimated, which is not addressed by the authors. Authors also offer interesting predictions regarding risk factors and non-pharmacological treatments for neuropathic pain.

Major points:

1. It’s important to stress that authors’ modelling results are based only on simulation data and theoretical representation (based on others’ findings in the literature discussed) of what constitutes placebo, nocebo and neuropathic pain effects. From first reading of the paper, this wasn’t clear to me, and I think it should be emphasised better. Given that authors propose a new way to update the parameters of the inference systems in the context of pain, it would be more convincing to apply the model to pre-existing datasets (for example Jepma et al. 2018 that authors cite, has a publicly accessible dataset: <https://osf.io/bqkz3/>) and plot model predictions and simulations alongside actual data. Particularly, this could be used to update Figure 1. Based on the figure caption, it’s not clear whether what’s plotted are results of the simulation using the model or a just a graphical aid to represent the concept of nocebo/placebo. Moreover, based on other modelling approaches (e.g. Jepma et al. 2018), participants’ pain ratings as well as associated model parameters tend to display a significant level of variability, as such it would be important to establish whether the proposed framework is able to capture some of this individual variability.

Minor points:

1) The authors outline many important questions in the introduction and mention an open question in the abstract. While I can mostly infer which question the authors tackle based on reading the entire manuscript, it would be helpful to explicitly state the question authors address in this work - it might just be a matter of rephrasing some of the existing sentences.
2) Regarding the author’s summary, I wouldn’t say that placebo/nocebo are counterintuitive.
3) Regarding the effects of increased uncertainty on experienced level of pain (Figure 3, 5), authors may considering interpreting their results alongside corroborating results from Yoshida et al. 2013(<https://doi.org/10.1523/JNEUROSCI.4984-12.2013>).
4) I’d recommend describing exactly what we see in the figures, where are the results coming from. E.g. “Results of the Kalman Filter simulations”, where tissue damage (line-type), expected/perceived pain (colour), I would also map these to model variable names.
5) More generally, figures would benefit from more attention. For example, in Figure 2, the legend items appear to be flipped (?). i.e I’d expect the red shaded line to correspond with perceived pain. Moreover, I’d suggest use of different colours/line-types to depict different values of A in Figure 3
6) Single layer KF model:
   a) The authors use a version of Kalman filter that allows for external (control) inputs to the system (u), which in the second paragraph of “Single-layer Kalman filter” section represent actions (u) and which are scaled by parameter B. It’s unclear what actions mean here. Especially when in the next paragraph, authors manipulate the values of B (B^{hat}) to account for the effects of brief noxious stimuli. In my understanding of the Kalman filter model, the “brief noxious stimuli” would be accounted for during the “update/measurement” step, where the prediction error (actual observation here brief noxious stimuli - predicted pain) is scaled by the Kalman gain. What is ‘real-world value of B’?
   b) Moreover, if the parameter A governs the dynamics of tissue damage, I take that “real-world value of A” means that A represents some biologically based parameter which actually represents tissue damage, while A^{hat}, would be participant’s approximation/estimate of this parameter, which in the case of placebo/nocebo phenomena would be lower/higher? If so, it would aid clarity to map the parameters a bit more precisely onto the pain system more.
7) HKF model:
   a) Figure 5: It’s difficult to parse out the relationship between A, A0, R parameters. Does the top panels show the inferred value of A over time? How do authors select A0 in the first place, as the value seems to have different starting points in top-right figure, but Table 2 would suggest they should start from the same value? Additional labelling/legends would be helpful to keep track of different cases and effects, but this all looks quite promising.
   b) Figure 6: A use of different colours or more clearly different line-types of A/B would be helpful to see differences in value. Additionally, it’s not clear how authors represent nerve injury (vertical dashes line) in the model? Is it through a sudden change in the uncertainty value R?
   c) “Our model corroborates that persistent non- neuropathic pain is a risk factor for developing neuropathic pain following denervation [28–30]”, I think this is an overstatement, however the authors offer a promising framework to investigate and model such risk factors in the population.
   d) Figure 7: Maybe change the caption where the first sentence describes what the reader can see rather than the description of offset analgesia, perhaps plot the true stimulus value at each time point vs HKF predictions?
   e) Is the scaling factor K needed? If R tends to infinity, then Kalman gain will approach 0, and so there will be no updates to the covariance and means of the estimated parameters. I’m not sure how scaling factor K addresses this? Additionally, try to keep the notation consistent through – scaling factor K is sometimes referred to as K and sometime as K^{(k)}.
8) Mention the simplifying assumption about H=1, in the single KF when the model is introduced in the methods rather than in the final section, otherwise the update equations for posterior estimates and Kalman gain are incorrect.
9) Given the above points about model description and clarity, I’d suggest an additional figure that depicts the evolution of states/external inputs and parameters of the model vs the environment in a style resembling a probabilistic model definition (see Jepma et al. 2018, Figure 5), with a clear description as to what each variable/parameter means and how it ties with the simulated variables and effects.
10) Regarding this statement "While such manually tuned models do give insight to how expectations can influence pain perception, they do not account for how previous experiences shape those expectations, and accordingly cannot explain phenomenon such as classical conditioning, offset analgesia or how certain events might predispose individuals to chronic or neuropathic pain.” - Authors need to be clearer about what ‘previous experiences’ mean. For example, some parameters in the Jepma et al. 2018 paper (confirmatory bias), could represent such “experiences” that shape expectations and in turn pain perception. I believe by ‘experience” authors meant different kinds of pain sequences (Figure 6) or nerve injury (Figure 5). If so, perhaps fleshing this out earlier would be advisable.
11) Update Figure reference in discussion: “ The simulation results presented in Figure X”.
12) This is too strong: “Thus, our model predicts that monotonous or paresthetic sensations of intermediate intensity might be more common in patients with partial denervation (𝑅 intermediate) while pain attacks, monotonously high intensity or complete absence of pain would be expected in patients with complete denervation (𝑅 high).” Perhaps say something along the lines: “the model offers a framework to understand why patients with partial denervation … “
13) Not sure how much we need a discussion about predictive coding, but I leave this decision to the authors. Again, the emotional experience of pain is highly interesting, however it seems outside the scope of this paper and the proposed model. I would consider removing to make the discussion to the point. Similarly, I would not discuss prospect theory in such a detail here. Perhaps package all the alternative models and ways to extend the modelling in 1-2 brief paragraphs.
14) There are some typos and phrasings throughout the manuscript that would benefit from additional proofreading to improve readability and add clarity.
