# Supplementary material for: A Computational Framework for Understanding the Impact of Prior Experiences on Pain Perception and Neuropathic Pain
Source: PLoS Comput Biol. 2024 Oct 31;20(10):e1012097. doi: 10.1371/journal.pcbi.1012097 (PMC11556707; doi:10.1371/journal.pcbi.1012097)
Supplement: Attachment — Submitted filename: Response to reviewers.docx [file pcbi.1012097.s003.docx]

Dear Section Editor Thomas Serre, Guest Editor Flavia Mancini, and reviewers,

We truly appreciate all the reviewers’ comments and their notable engagement in reviewing our work. We believe that the comments identified limitations in our original manuscript and addressing these comments has helped us to improve our manuscript significantly. To facilitate the review of our responses and actions taken, we are including a formatted file with the comments from the editors and reviewers in italic, each followed by our responses and actions colored in blue. We are providing two revised files of the manuscript (one with tracked changes and one with all changes accepted).

### Comment from the editors

*The guest editor and reviewers acknowledge the novelty and importance of providing a theoretical framework to describe pain perception and its modulation. The reviewers have raised a number of major and minor points that would warrant a major revision. The main areas of concern are:*

*1) Clarity, especially regarding the explanation of modelling choices and results*

Action: We have conducted a thorough review of the model based on the feedback from the reviewers and updated the description of the model accordingly. We have made a concerted effort to the improve the clarity in our description and explanations of modelling choices and results. These updates have been implemented throughout the manuscript, with particular emphasis in the Results and Methods sections.

*2) Modelling - The simulation results could be validated by fitting existing, open datasets (see Reviewer 1 comments)*

Response: We found this input from Reviewer 1 to be very valuable. Validating our results with the suggested dataset improves the credibility of our model results.

Action: The model parameters have been tuned to give output that matches the experimental data from the suggested dataset. In Figure 1 we plot the model results alongside the experimental data, to make comparison easy for the reader. For Figure 4 we simulated the experimental paradigm that was used to produce the dataset to show that our model not only can produce results that align with the experimental data, but also can account for the process leading up to those results. Descriptions of the relevant simulations have been updated in the Methods section.

*3) The conceptual definition of a number of terms (see Reviewer 2 comments)*

Action: In revising the model and the manuscript we have made a few conceptual updates, in part due to this feedback. We have taken special care to describe and justify our modelling choices in the manuscript, particularly in the Methods and the Results sections.

### Reviewer 1

*Authors highlight an open question in the field of pain neuroscience as to what mechanisms/processes gives rise to prior beliefs, which in turn affect the experienced level of pain, with relevance to chronic pain states but also effects such as placebo and nocebo. This is of course a very important issue as we know surprisingly little about pain inference, especially in the times where chronic pain is such a prevalent issue (20-30% of population). Authors briefly outline the relevant research to position their question within, particularly mentioning Bayesian inference as a promising way to model such phenomena. Authors themselves propose the use of a (modified) Kalman Filter (KF) model (an algorithm used to estimate latent states in a specific setup of probabilistic / Bayesian inference) to describe phenomena such as placebo hypoalgesia, nocebo hyperalgesia and neuropathic pain.*

*The authors indeed succeed in reproducing the stated phenomena (placebo/nocebo, neuropathic pain) through simulation. They also demonstrate their model’s predictions in the context of classical conditioning paradigm to induce nocebo/placebo effects. The results are interesting and it’s promising that the HKF model is able to capture these effects. However, in order to appreciate this, the reader is required to jump between figures captions, main texts and methods to put a story together. As such, the article will benefit from more clarity regarding modelling descriptions and results reporting (with a clearer mapping between the two). Additionally, a comparison of model predictions to a pre-existing dataset (see comment below) could provide a more convincing story. The proposed application of Hierarchical Kalman Filter in the context of pain is novel and holds promise for modelling pain inference. Additionally, it allows for some of the system parameters to be estimated online “by the participant’s sensory/cognitive system” rather than inferred post-hoc from the data, and as such is a valuable approach. However, it’s important to note that other parameters still need to be hand-tuned or estimated, which is not addressed by the authors. Authors also offer interesting predictions regarding risk factors and non-pharmacological treatments for neuropathic pain.*

Response: Thank you for your valuable feedback on the work presented in this manuscript. We appreciate your recognition of the novelty of our work, as well as your identification of its limitations and the constructive feedback you provided to help us improve our model and manuscript. The suggestion of comparing the model predictions to experimental data and directing us to exactly such an open-source dataset has been very helpful.

Actions: We have conducted a thorough review of the manuscript. We have made a concerted effort to the improve the clarity in our description and explanations of modelling choices and results and the structure of the manuscript to improve readability (while still complying with the author guidelines provided by the journal).

We have updated the simulations relating to placebo hypoalgesia and nocebo hyperalgesia to allow for comparison with the suggested dataset (see Figure 1 and Figure 4).

The point on parameter estimation is very important. We have now included this in our discussion, see paragraph 6 in the Discussion section.

*Major points:*

1. *It’s important to stress that authors’ modelling results are based only on simulation data and theoretical representation (based on others’ findings in the literature discussed) of what constitutes placebo, nocebo and neuropathic pain effects. From first reading of the paper, this wasn’t clear to me, and I think it should be emphasised better. Given that authors propose a new way to update the parameters of the inference systems in the context of pain, it would be more convincing to apply the model to pre-existing datasets (for example Jepma et al. 2018 that authors cite, has a publicly accessible dataset:* [*https://osf.io/bqkz3/*](https://osf.io/bqkz3/)*) and plot model predictions and simulations alongside actual data. Particularly, this could be used to update Figure 1. Based on the figure caption, it’s not clear whether what’s plotted are results of the simulation using the model or a just a graphical aid to represent the concept of nocebo/placebo. Moreover, based on other modelling approaches (e.g. Jepma et al. 2018), participants’ pain ratings as well as associated model parameters tend to display a significant level of variability, as such it would be important to establish whether the proposed framework is able to capture some of this individual variability.*

Response: Thank you for this valuable input. We agree that the model results could be given more credibility if they are verified with experimental data. We had overlooked the opportunity of using the publicly accessible data from (Jepma et al., 2018) and appreciate you bringing this to our attention.

Actions: We have used the suggested dataset to tune the model parameters to give output that matches the experimental data. Figure 1 and the associated simulations have been updated to plot our model predictions along with the experimental data from (Jepma et al., 2018). We have also updated our simulated classical conditioning to match the experimental paradigm described by (Jepma et al., 2018), allowing these results to also be comparable to the experimental data. The methodological changes are described in brief in the Results section and more extensively in the Methods section.

The updated simulations reveal that our models, both the traditional Kalman filter and the modified hierarchical Kalman filter, indeed do capture some of the individual variability in expected and perceived pain. While the simulated and experimental data are qualitatively similar, we acknowledge that there are quantitative differences. These discrepancies are discussed in the Discussion section, paragraph 6.

We have tried to clarify in the Introduction that the placebo/nocebo results are verified by experimental data, and that the rest of the results are based on simulations. These updates can be found in the last paragraph of the introduction.

*Minor points:*

1) *The authors outline many important questions in the introduction and mention an open question in the abstract. While I can mostly infer which question the authors tackle based on reading the entire manuscript, it would be helpful to explicitly state the question authors address in this work - it might just be a matter of rephrasing some of the existing sentences.*

Action: Thank you for pointing this out. We have rephrased of the last paragraph in the introduction to clarify the objectives of the research and what questions are being addressed.

2) *Regarding the author’s summary, I wouldn’t say that placebo/nocebo are counterintuitive.*

Response: The author’s summary is intended to for a wide audience. While placebo/nocebo effects might not seem counterintuitive if one is familiar with the complexities of sensory perception, we believe others may find the phenomenon in which expectations can alter the level of experienced pain to be counterintuitive.

3) *Regarding the effects of increased uncertainty on experienced level of pain (Figure 3, 5), authors may considering interpreting their results alongside corroborating results from Yoshida et al. 2013(*[*https://doi.org/10.1523/JNEUROSCI.4984-12.2013*](https://doi.org/10.1523/JNEUROSCI.4984-12.2013)*).*

Action: Thank you for bringing this resource to our attention, it is highly relevant for our work and very interesting. From our understanding, (Yoshida et al., 2013) study the uncertainty in the cues that inform the prior estimate, and how this uncertainty influences the perceived pain. Based on how we have defined our model, this uncertainty would correspond to the value of $Q$. In Figures 3 and 5 we focus on the influence of the uncertainty on the sensory input, which corresponds to the variable $R$. Nevertheless, the results by (Yoshida et al., 2013) are relevant to our work and offer an interesting avenue for future use and development of the model we have presented. We have included reflections on (Yoshida et al., 2013) in the Discussion section, paragraph 4.

4) *I’d recommend describing exactly what we see in the figures, where are the results coming from. E.g. “Results of the Kalman Filter simulations”, where tissue damage (line-type), expected/perceived pain (colour), I would also map these to model variable names.*

Response: Thank you for this helpful suggestion. Figure captions have been updated to describe the content of the figures and disclose if it is simulation results or experimental data that are being presented more clearly. Descriptions of the graphed variables and mapping to model variable names have been added to the captions.

5) *More generally, figures would benefit from more attention. For example, in Figure 2, the legend items appear to be flipped (?). i.e I’d expect the red shaded line to correspond with perceived pain. Moreover, I’d suggest use of different colours/line-types to depict different values of A in Figure 3*

Response: Thank you for bringing the error in Figure 2 to our attention. The legend in Figure 2 has been updated. Colors and line width have been adjusted in Figure 3 and 5 to make the lines for different values of A more distinct.

6) *Single layer KF model:*
   a) *The authors use a version of Kalman filter that allows for external (control) inputs to the system (u), which in the second paragraph of “Single-layer Kalman filter” section represent actions (u) and which are scaled by parameter B. It’s unclear what actions mean here. Especially when in the next paragraph, authors manipulate the values of B (B^{hat}) to account for the effects of brief noxious stimuli. In my understanding of the Kalman filter model, the “brief noxious stimuli” would be accounted for during the “update/measurement” step, where the prediction error (actual observation here brief noxious stimuli - predicted pain) is scaled by the Kalman gain. What is ‘real-world value of B’?*

Response: In the updated manuscript we have revised the model parameter definitions. Specifically, we have changed the definition of the control input ($u^{\left( k \right)}$) to be “factors that may predict changes in the perceived level of pain”. This revised definition allows for the inclusion of factors such as motor actions, environmental context and cues, and possibly psychological processes such as thoughts and emotions. This definition of control input differs somewhat from how control input is typically defined in control theory and engineering applications, where this term often refers to an external input or force that is deliberately applied to the system to influence its behavior or state. The traditional definition of control input would be applicable if we were interested in modelling the actual tissue damage, but that is not the goal of this model. Instead, we are trying to model how the brain models tissue damage. As such, $\hat{B}$ would reflect the expected gain in tissue damage from control input $u^{\left( k \right)}$.

In some of our simulations we do model tissue damage as $x^{\left( k \right)}=Ax^{\left( k-1 \right)}+B\tilde{u}^{\left( k \right)}$, to allow for comparison between the level of true tissue damage and the perceived level of pain (see Figure 3 and Figure 6). In these simulations $\tilde{u}^{\left( k \right)}$ corresponds to input that influences the level of tissue damage, such as force or heat applied to the tissue. Sometimes there is a strong correlation between $\tilde{u}^{\left( k \right)}$ and $u^{\left( k \right)}$, for example if the tissue damage is caused by a motor action, or if there are visual cues predictive of the imminent tissue damage. Other times $\tilde{u}^{\left( k \right)}$ and $u^{\left( k \right)}$ may be completely independent, such as unknowingly being stung by a bee. In these simulations of tissue damage the value of $B$ reflects the gain in tissue damage from input $\tilde{u}^{\left( k \right)}$.

Action: Changes in the model definitions, and clarifications on what the model parameters represent, have been implemented in the Methods and Results sections.

b) *Moreover, if the parameter A governs the dynamics of tissue damage, I take that “real-world value of A” means that A represents some biologically based parameter which actually represents tissue damage, while A^{hat}, would be participant’s approximation/estimate of this parameter, which in the case of placebo/nocebo phenomena would be lower/higher? If so, it would aid clarity to map the parameters a bit more precisely onto the pain system more.*

Response: Yes, in the simulations where we model tissue damage as $x^{\left( k \right)}=Ax^{\left( k-1 \right)}+B\tilde{u}^{\left( k \right)}$, the parameter $A$ governs the dynamics of actual or potential tissue damage in absence of external input and would thus encompass factors such as healing/recovery of baseline tissue function and/or degradation of tissue state through e.g., inflammation. As for placebo/nocebo, in our model this effect is reflected in the value of $\hat{B}$, as $\hat{B}$ links the conditioned stimuli to the expected level of pain. In the revised manuscript we have updated the simulations of placebo/nocebo such that each trial is treated as a discrete point in time, rather than simulating the temporal dynamics within and between trials. This approach allows us to further simplify the model and exclude $\hat{A}$ in these simulations.

Action: Changes in the model definitions, and clarifications on how the model parameters map onto the pain system, have been implemented in the Methods and Results sections.

7) *HKF model:*
   a) *Figure 5: It’s difficult to parse out the relationship between A, A0, R parameters. Does the top panels show the inferred value of A over time? How do authors select A0 in the first place, as the value seems to have different starting points in top-right figure, but Table 2 would suggest they should start from the same value? Additional labelling/legends would be helpful to keep track of different cases and effects, but this all looks quite promising.*

Action: In addition to a general revision of all figure captions, the caption of Figure 5 has been updated to better describe the graphs and to include clarification of the different initial values $\hat{A}_{0}$.

b) *Figure 6: A use of different colours or more clearly different line-types of A/B would be helpful to see differences in value. Additionally, it’s not clear how authors represent nerve injury (vertical dashes line) in the model? Is it through a sudden change in the uncertainty value R?*

Action: Yes, nerve injury is represented by an increase in R. Caption of figure 6 has been updated to use different colors and line-types for $\hat{A}$ and $\hat{B}$ and with a clarification that nerve injury corresponds to a change in the value of R and is indicated by the vertical dashed line

c) *“Our model corroborates that persistent non- neuropathic pain is a risk factor for developing neuropathic pain following denervation [28–30]”, I think this is an overstatement, however the authors offer a promising framework to investigate and model such risk factors in the population.*

Action: This sentence has been reworded.

d) *Figure 7: Maybe change the caption where the first sentence describes what the reader can see rather than the description of offset analgesia, perhaps plot the true stimulus value at each time point vs HKF predictions?*

Response: We find that plotting the true tissue damage (which is what we assume is being referred to as ‘true stimulus’) to make the graph crowded and more difficult to read. Furthermore, we believe that the interesting comparison is between the perceived pain in the Offset analgesia and control conditions, rather than comparing the pain to the true tissue damage. Finally, the current format of the graph allows for straight forward comparison with how experimental results have been presented elsewhere, e.g., by (Grill & Coghill, 2002): <https://doi.org/10.1152/jn.00730.2001>.

Action: Caption of Figure 7 has been updated to give a clearer description of what is shown in the figure.

e) *Is the scaling factor K needed? If R tends to infinity, then Kalman gain will approach 0, and so there will be no updates to the covariance and means of the estimated parameters. I’m not sure how scaling factor K addresses this? Additionally, try to keep the notation consistent through – scaling factor K is sometimes referred to as K and sometime as K^{(k)}.*

Response: The scaling factor $K^{\left( k \right)}$ is the Kalman gain of the first/state estimation layer. Our reason for including $K^{\left( k \right)}$ in the update of $P_{p}$ is motivated by probing intuitive extremes. As you have already pointed out, if $R = \infty$, the parameters are not updated. However, though $K_{p}^{\left( k \right)}$ is influenced by $P^{\left( k \right)}$, it does not go to 0 when $R = \infty$. Thus, if the covariance of the parameter update, $P_{p}^{\left( k+1 \right)}$, is only modulated by $K_{p}^{\left( k+1 \right)}$ it will decrease, indicating that the uncertainty in the parameter estimate is decreasing. One should of course not become more confident in one’s parameter estimate if there is infinite sensory noise. In contrast, modulating by $K^{\left( k \right)}K_{p}^{\left( k+1 \right)}$ does have the desired effect of ensuring that $P_{p}^{\left( k+1 \right)}$ does not decrease when $R = \infty$.

Action: Notation has been updated throughout the manuscript to include the temporal index of model parameters in superscript where it is relevant for the context. Occasionally, where the temporal index is not important for the context, we refer to model parameters without the index to improve readability of the text.

8) *Mention the simplifying assumption about H=1, in the single KF when the model is introduced in the methods rather than in the final section, otherwise the update equations for posterior estimates and Kalman gain are incorrect.*

Action: Thank you for bringing this to our attention. The assumption about $H=1$ is now included immediately after $H$ is first mentioned in the single KF section.

9) *Given the above points about model description and clarity, I’d suggest an additional figure that depicts the evolution of states/external inputs and parameters of the model vs the environment in a style resembling a probabilistic model definition (see Jepma et al. 2018, Figure 5), with a clear description as to what each variable/parameter means and how it ties with the simulated variables and effects.*

Response: This is an excellent suggestion. We have now included schematic figures of both the traditional Kalman filter model and the hierarchical model. Hopefully they can bring some more clarity to the model description.

Action: Schematic figures of the models have been added as Figure 8 and Figure 9.

10) Regarding this statement "While such manually tuned models do give insight to how expectations can influence pain perception, they do not account for how previous experiences shape those expectations, and accordingly cannot explain phenomenon such as classical conditioning, offset analgesia or how certain events might predispose individuals to chronic or neuropathic pain.” - Authors need to be clearer about what ‘previous experiences’ mean. For example, some parameters in the Jepma et al. 2018 paper (confirmatory bias), could represent such “experiences” that shape expectations and in turn pain perception. I believe by ‘experience” authors meant different kinds of pain sequences (Figure 6) or nerve injury (Figure 5). If so, perhaps fleshing this out earlier would be advisable.

Response: We appreciate your input on this point. In our reading, the confirmatory bias in (Jepma et al., 2018) can be viewed as *modulating* expectations, by enforcing stronger expectation updating when new experiences are consistent with the initial expectation of high or low pain. While this does reflect some aspects of how previous experiences *shape* future expectations, the model does not account for how the initial expectations *arise* from the learning phase of the experimental paradigm.

Action: We have rephrased the statement to more accurately specify that previous models “do not account for how previous experiences *give rise* to expectations”. Furthermore, we have clarified that it is “the context and dynamics of previous pain experiences” that is of particular interest for our model.

11) *Update Figure reference in discussion: “ The simulation results presented in Figure X”.*

Action: Thank you for bringing this to our attention. The figure reference has been updated to “Figure 5”.

12) *This is too strong: “Thus, our model predicts that monotonous or paresthetic sensations of intermediate intensity might be more common in patients with partial denervation (𝑅 intermediate) while pain attacks, monotonously high intensity or complete absence of pain would be expected in patients with complete denervation (𝑅 high).” Perhaps say something along the lines: “the model offers a framework to understand why patients with partial denervation … “*

Response: Here, we are describing how the model output might map onto various clinical profiles. However, we have not found any empirical data to verify the predicted relationship between level of denervation and pain characteristics.

Action: Changed “predicts” to “suggests” and added a sentence at the end of the paragraph to emphasize that the predictions are yet to be validated by empirical data.

13) *Not sure how much we need a discussion about predictive coding, but I leave this decision to the authors. Again, the emotional experience of pain is highly interesting, however it seems outside the scope of this paper and the proposed model. I would consider removing to make the discussion to the point. Similarly, I would not discuss prospect theory in such a detail here. Perhaps package all the alternative models and ways to extend the modelling in 1-2 brief paragraphs.*

Action: The paragraph on predictive coding has been removed, as multiple reviewers had similar comments. The discussion on ways to extend the model has been shortened, leaving out the details on prospect theory.

14) *There are some typos and phrasings throughout the manuscript that would benefit from additional proofreading to improve readability and add clarity.*

Action: In revising the manuscript, we have done our best to address any typos, grammatical errors or confusing phrasing to improve the readability and clarity of the text.

### Reviewer 2

*In the manuscript titled "A Computational Framework for Understanding the Impact of Prior Experiences on Pain Perception and Neuropathic Pain", authors Ramne and Sensinger expand a Bayesian approach (single-layer Kalman filter) of modelling pain genesis to also include parameter updates over time, using a second layer for hierarchical Kalman filtering. This allows for consideration of _concurrent processes_ like expectation, as well as the _formation_ of the respective process parameters via previous experiences. Their models (partially) replicate/mimick several known physiological & pathological pain phenomena, including expectancy-based & conditioning-based placebo/nocebo effects, offset analgesia, chronic pain & neuropathic pain arising from increased sensory uncertainty. The article is well-written and the methods are intriguing, and the approach could provide an overall useful tool (for computational modelling, obviously, but also for hypothesis generation and detecting commonalities and differences between pain entities). However, I would contest some imprecisions and limitations of the models, as well as point out general shortcomings of the manuscript. Many of those points are not "this is wrong" but rather "please consider".*

Response: Thank you for your valuable feedback on our manuscript. We appreciate your recognition of the novelty of our work, as well as your identification of its limitations and the constructive suggestions you provided to help us improve both our model and the manuscript.

*I. MAJOR ISSUES
[for the first two comments I have no good suggestion on how to deal with them but I encourage the authors to work on these parts; if there was a more coherent characterization of the parameters of the models, readability for a wider audience might improve]

I.1. The authors consider both "actual and potential tissue damage" for noxious input, as per IASP definition. I have a semantic and a conceptual concern about the current framing: Semantically, the current characterization of parameter A (or ^A, respectively) is a reference to "healing", but if only potential tissue damage takes place (say, transient experimental pain without actual injury), what does "healing" refer to? Maybe "recovery of baseline function" or some such formula is a more suitable description, as it could pertain to compensation or recovery (recovered tissue integrity, or recovered sensitivity, or even loss of memory of the painful episode). Conceptually, the authors do point out that the level of abstraction of the models is rather high, i.e. does not posit any particular implementation or mechanism, just describes the outcome of whatever mechanism might be at work. Ultimately, I would like them to point out that there are overlapping and distinct mechanisms at work depending on the specific pain that is experienced.*

Response: Thank you for your observant and well formulated feedback. In the revised manuscript we have made some conceptual updates to the model that we think address this point. In the revised manuscript we emphasize that we are *not* attempting to provide a precise model the dynamics of tissue damage, but rather a model of *how the brain models* tissue damage. As such, $\hat{A}\hat{x}^{\left( k-1 \right)}+\hat{B}\boldsymbol{u}^{\left( k \right)}$ is the brain’s model of how (actual or potential) tissue damage develops over time, resulting in an expected level of pain, ${\hat{\bar{x}}}^{\left( k \right)}$. Thus, $\hat{A}$ reflects how the brain expects pain to develop in absence of control input, where $0<\hat{A}<1$ reflects that pain is expected to diminish, and $\hat{A}>1$ reflects pain is expected to worsen with time.

Action: In the first paragraph of the methods section, we now point out that “the level of tissue damage is influenced by a wide range of overlapping and distinct mechanisms depending on the specific pain that is experienced”. Throughout the manuscript we have updated our specification and description of the model. These changes are most prominent in the Methods and Results sections.

*I.2. Relatedly, from a psychological perspective, I am not satisfied with the descriptions for processes at work during time points k and k-1, namely "actions" Bu and "healing" A (see above). At its core, Bu(k) and Ax(k-1) agnostically refer to _all processes pertaining to the respective time points k or k-1_ (however weighted). Colloquially and in some psychological settings, "action" would refer to overt and deliberate actions; while it is fine (and possibly conventional) to use the word in this case, I would at least clarify that it can pertain to any processes (e.g. neuropsychological processes like attention/distraction, physiological processes, motor behaviors; also, although the authors later suggest this is not the case, descending modulation). This is hinted at in the methods section but would clarify things if it was mentioned earlier.*

Response: In revising the model and manuscript, one of the key conceptual changes we have made pertains to the control input. We now define control input, $u$, as “factors that may predict changes in the perceived level of pain”. This revised definition allows for the inclusion of factors such as motor actions, environmental context and cues, and possibly psychological processes such as thoughts and emotions. This definition of control input differs somewhat from how control input is typically defined in control theory and engineering applications, where this term often refers to an external input or force that is deliberately applied to the system to influence its behavior or state. The traditional definition of control input would be applicable if we were interested in modelling the actual tissue damage, but that is not the goal of this model. Instead, we are trying to model how the brain models tissue damage. As such, $\hat{A}$ reflects how the brain expects pain to develop in absence of control input and $\hat{B}$ reflects the expected gain in tissue damage from control input $u$.

While we are not trying to model tissue damage, but rather how the brain models tissue damage, in some of our simulations we do model tissue damage as $x^{\left( k \right)}=Ax^{\left( k-1 \right)}+B\tilde{u}^{\left( k \right)}$, to allow for comparison between the level of true tissue damage and the perceived level of pain (see Figure 3 and Figure 6). In these simulations, $A$, $B$ and $\tilde{u}^{\left( k \right)}$ indeed do reflect all processes pertaining to tissue damage, e.g., external stimuli, motor actions and physiological processes such as healing and inflammation.

*I.3 Line 244. "First, we show that there is no expectation of pain associated with the conditioned stimulus at baseline." I was having trouble grasping how this was meant. In the design implicit in the sequence of events of Figure 4, the results would indicate an _expectation of no/zero pain_ (from the zero pain applied at T1/Baseline, which "pulls down" the expectation initially), not _no expectation_. In practice, this is not how the CS is introduced as this would probably lead to latent inhibition. Instead, the CS being neutral indicates that is it conditionable, not that it is conditioned to zero pain. Put bluntly, in placebo conditioning, the CS is not shown with the baseline stimulation (besides, "zero pain" is not a baseline condition. I am not sure how to amend this model-wise, but I feel the placebo/nocebo conditioning part is unconvincing as is.*

Response: The simulations of classical conditioning have been extensively revised in the updated submission. Another reviewer directed us to an open-source dataset with experimental data from a placebo/nocebo experiment (<https://osf.io/bqkz3/>). In addition to expected and perceived pain for cues that have been conditioned to be associated with high or low pain, the dataset includes ratings for neutral cues that were not included in the conditioning phase of the experiment. As you point out, these neutral cues are not associated with zero pain. We used the expected pain rating for the neutral cues to inform the baseline value of $\hat{B}$, which determines the expectation of pain associated with the cue, in our updated simulations of classical conditioning.

*I.4 Line 249ff. From what I see, the nocebo results do _not_ match the single-layer Kalman results in Fig 1, not even "qualitatively". This may be related to deficiencies in the model pointed out in I.3; plot inspection indicates that the conditioning procedure never overcame the "zero pain" conditioning from the baseline but still produced a placebo effect despite (ostensibly) nocebo conditioning. The statement that there is replication of placebo/nocebo conditioning seems unwarranted at this stage.*

Response: Thank you for bringing this to our attention. Somewhere in the process of updating code and figures, the similarity in the placebo/nocebo effect obtained with the two different Kalman models was lost.

Action: These simulations have been extensively revised in the updated manuscript, and importantly are now comparable to experimental data. Crucially, Figure 1 and Figure 4 now do show qualitatively similar patterns. Quantitative differences between the simulations and the experimental data are discussed in the Discussion section, paragraph 6.

*II. MINOR ISSUES (semantic & conceptual)
- The first paragraph of the Results have an Introductory feel to them.*

Response: We see now that most of the information in the first paragraph of the Results had already been covered in the introduction. As for the next few paragraphs, they are inteded to provide background on the methods used, specifically for readers who may not be familiar with Bayesian models. Given that the Results section precedes the Methods in this journal, our goal is to offer sufficient context here to help readers understand and interpret the results.

Action: We have moved a few sentences from the first paragraph of the Results to the third paragraph of the Introduction, and removed the rest to avoid redundantly repeating information.

*- Line 133 "intensity, quality, and characteristics" - are intensity and quality not characteristics? Maybe an "other" is missing here...*

Action: Yes, of course. Thank you for pointing this. This sentence has been amended as “intensity, quality, and other characteristics…”

*- Line 135ff. At first reading, I found the elaboration of the formulae in the Results section a bit lacking (possibly owing to the imho unfortunate PLOS-sided postponing of the Methods). k for example is not defined.*

Response: We have tried to limit the formulae and method descriptions in the Results section as much as possible, while still providing enough context to make the results understandable.

Action: Definition of $k$ has been added to the first paragraph of the Results.

*- Figure 2. "True tissue damage" may be more suitably described as "nociceptive signal" or "afferent drive" (see I.1)?*

Response: The way we have defined the model, tissue damage and nociceptive signaling are distinct from each other, where $x$ corresponds to true tissue damage and $z=Hx+ \epsilon(0,R)$ corresponds to the afferent nociceptive signals.

*- Figure 2. Your legend entries were flipped for the second row - solid line is perceived pain, dotted line is "true tissue damage" (or whatever alternative). Incidentally, MATLAB warns that you provide extra legend entries (using the uploaded code).*

Action: Thank you for bringing this to our attention. Legend entries have been corrected in the figure.

*- Line 183, "In the context of Bayesian models, elevated uncertainty of sensory input is a requirement for expectations to have a significant enough influence that they cause the level of pain to deviate from what is signaled by the sensory input." Very vague statement, why does uncertainty have to be "elevated" and what is "significant enough" or "deviation"? Maybe rephrase to point to the fact that _the more_ likelihood uncertainty and _the less_ prior uncertainty, the more the posterior will correspond to the prior, or so? Anyway please put more concisely.*

Response: We see now that this sentence may have been somewhat confusing.

Action: The first few sentences of the paragraph have been updated to more accurately describe how uncertainty in the prior and likelihood affect the model output.

*- Line 193, "The gradual increase of pain before the onset of tissue damage in the bottom right panel of Figure 2" - does this refer to the ~0.1 point increase visible in the graph? I'm not convinced that would constitute "how pain can arise spontaneously" (given the dispersion that makes any increase in that range rather cosmetic), and I don't think your message would be impacted if you did not provide this Figure 2 observation to introduce Figure 3.*

Response: This refers to the bottom right panel of Figure 2, where there is a ~3 point increase in perceived pain.

*- Figure 3. I would find this figure much more informative if you used Rs = [1 10 100]*sKFParams.R; instead of Rs = [1 100 1e6]*sKFParams.R; That way, you could demonstrate that the model does not simply produce ceiling effects, and I don't recall reading that you discussed the initial "overshoot" in the R=600² condition anyway. I appreciate though that for Figure 5 you want to preserve R=600², but four panels also wouldn't hurt as the individual panels are not overloaded with information anyway.*

Action: Figure 3 has been updated to plot four panels with R=[1 100 1e4 1e6]* sKFParams.R = [${0.8}^{2}$, $8^{2}$, ${80}^{2}$, ${800}^{2}$]. The initial overshoot for $\hat{A}=1.1$ and $R=[{80}^{2}, {800}^{2}]$ is commented in the figure caption.

*- Line 236ff. I would add that the conditioned response in placebo/nocebo conditioning would be the processes accompanying the painful stimulus, presumably "the state anticipating a noxious stimulus of intensity x" (possibly even "psychomotoric readiness to give rating in a certain range of the scale", but that might be a bit iconoclastic).*

Action: We added a sentence to the paragraph on classical conditioning to clarify to clarify the what the conditioned response corresponds to in the context of placebo hypoalgesia and placebo hyperalgesia.

- Discussion could be more conscise, maybe skip the detour to predictive coding (I realize that this paragraph may have been desired by a different reviewer and I'm okay with it, just making the observation that the discussion feels a tad too long).

Action: The paragraph on predictive coding has been removed, as multiple reviewers had similar comments. We have also made an effort to shorten to be more concise in the discussion.

*- Line 364 "To our awareness, this phenomenon has not previously been reproduced in mathematical models of pain." - it has (Cecchi et al, PLOS Comput Biol 2012), and I do recommend checking out their model (using derivatives and many more parameters) as it provides very good approximation to OA (and transient/phasic pain stimulation in general).*

Response: Thank you for bringing this model to our attention.

Action: We have taken out the sentence on OA not being demonstrated by previous model. We have also included this reference in our discussion of limitations that might be contributing to our offset analgesia simulations producing an effect that is smaller than typically observed in experiments or demonstrated in the (Cecchi et al., 2012) model.

*- Line 448 "This limitation also means that our model cannot account for pain phenomenon that may arises because of impairment of pain modulation, such as certain forms of chronic pain." Wouldn't Bu(k) account for descending modulation? (see I.2)*

Response: This is an interesting perspective. Depending on how sensory and control input are defined in specific implementations of the model, certain forms of descending pain modulation indeed could be accounted for by $\hat{B}u$. However, we also believe that there are a number of pain modulatory mechanisms that cannot be captured with the model in its current form.

Action: We have nuanced this sentence to: “This limitation also means that our model may be limited in describing pain phenomena that arise because of impairment of certain pain modulatory mechanisms, as is the case in certain forms of chronic pain.”

*- Line 471. I would contest that allodynia describes a behavioral outcome (non-noxious intensity causing pain), not the genesis of that outcome. I.e. allodynia can arise through multiple processes (sensitiziation, inflammation, etc.) and is not solely a neuropathic phenomenon. Please revise.*

*Action: The word “neuropathic” has been removed from the sentence.*

*- Line 518ff. Hm I'm not that sold on prospect theory, the paragraph also seems to conflate appetitive and aversive motivation in regards to pain, which follow different functions (loss = usually losing something good, not = gaining something bad)... is it possible to find a more concrete/analogous application in the aversive domain?*

Action: This paragraph has been removed in an effort to shorten the discussion, as requested by multiple reviewers.

*- Line 660. Replace "commonly" with "frequently".*

Action: “commonly” has been replaced with “frequently”.

*III. MINOR ISSUES (formal)
- Figure 2. Your legend here says "True tissue damage" but all other figures use "Tissue damage".
- Line 423. "electric shocks" => "electric-shock-like sensations"
- Line 423. "The simulation results presented in Figure X indicate that moderately high values or R"... (Figure X => Figure ?, or => of)
- Line 448 "pain phenomenon that may arises"
- Line 481 "it seemingly does to provide pain relief"
- Line 532 "concepts form prospect theory"
- Line 661 "exist on similar 11-point scale"
- References: Some of the references have been mixed up (16==25, 26==49)*

Response: Thank you for bringing these issues to our attention.

Action: The listed issues have been attended to. Unfortunately, some of the references are still duplicated in the Word-file with tracked changes due to issues with the reference managing software. The references should be better organized in the clean version.

*IV. CODE ISSUES
- Possibly a MATLAB version issue, but mine throws an error at PlotFigure6:116
% scatter(iEvent, .5, 200, 'filled', 'pentagram', 'MarkerFaceColor',[0.4667 0.6745 0.1882]);
% this should probably be
scatter(iEvent, repmat(.5,size(iEvent)), 200, 'filled', 'pentagram', 'MarkerFaceColor',[0.4667 0.6745 0.1882]);*

Response: Thank you for bringing this issue to our attention. We cannot reproduce the error on our end but have updated the line of code to avoid errors in other versions of Matlab.

### Reviewer 3

*Well made manuscript. Recommend correcting minor grammatical mistakes.*

Response: Thank you. In revising the manuscript, we have tried to correct any grammatical errors. The manuscript has been reviewed by native-English speakers. If there are any more minor grammatical mistakes, we would be happy to fix them, but ask the reviewer to be more specific regarding their location and/or type.
